# Acute focal bacterial nephritis is associated with invasive diagnostic procedures - a cohort of 138 cases extracted through a systematic review

**DOI:** 10.1186/s12879-017-2336-6

**Published:** 2017-04-04

**Authors:** Nadine Sieger, Iason Kyriazis, Alexander Schaudinn, Panagiotis Kallidonis, Jochen Neuhaus, Evangelos N. Liatsikos, Roman Ganzer, Jens-Uwe Stolzenburg

**Affiliations:** 1 Department of Urology, University Hospital Leipzig, Liebigstraße 20, 04103 Leipzig, Germany; 2 Department of Urology, University Hospital of Patras, Rio 265 04, Patras, Greece; 3 Department of Diagnostic and Interventional Radiology, University Hospital Leipzig, Liebigstraße 20, 04103 Leipzig, Germany

**Keywords:** Focal nephritis, Renal infection, Upper urinary tract infection, Pyelonephritis

## Abstract

**Background:**

Acute focal bacterial nephritis (AFBN) is a rare disease currently described only in case reports and small case series. In this study we summarize the clinical features of AFBN as has been documented in the literature and draw recommendations on the proper diagnosis and therapy.

**Methods:**

A systematic literature review was undertaken in PUBMED, Web of Science and The Cochrane Library online databases for relevant literature on AFBN in adults.

**Results:**

Literature review revealed a total of 38 articles according to our inclusion criteria, of which we could extract data from 138 cases of AFBN. Fever (98%) and flank pain (80%) were most commonly reported symptoms. *E. coli* was the most frequent pathogen. Diagnosis was set by CT and/or MRI (52%) with or without sonography or by sonography alone (20%) as well as by sonography combined with IVU. In total, sonography was applied in 83% of cases. All but one patient received antibiotic treatment. Kidney lesions were occasionally mistaken for neoplasms or renal abscesses and as a result, cases were subjected to percutaneous puncture (12.3%), surgical exploration (5.1%) and partial or radical nephrectomy (4.4%). Four cases (2.9%) developed a renal abscess.

**Conclusions:**

The diagnosis of AFBN is set by characteristic clinico-radiological findings. Differential diagnoses of this interstitial bacterial infection include renal abscess and tumor. Correct diagnosis is occasionally impeded by atypical symptoms. Invasive diagnostic and therapeutic procedures should be limited as the majority of cases respond well to conservative treatment.

## Background

**Fig. 1:**
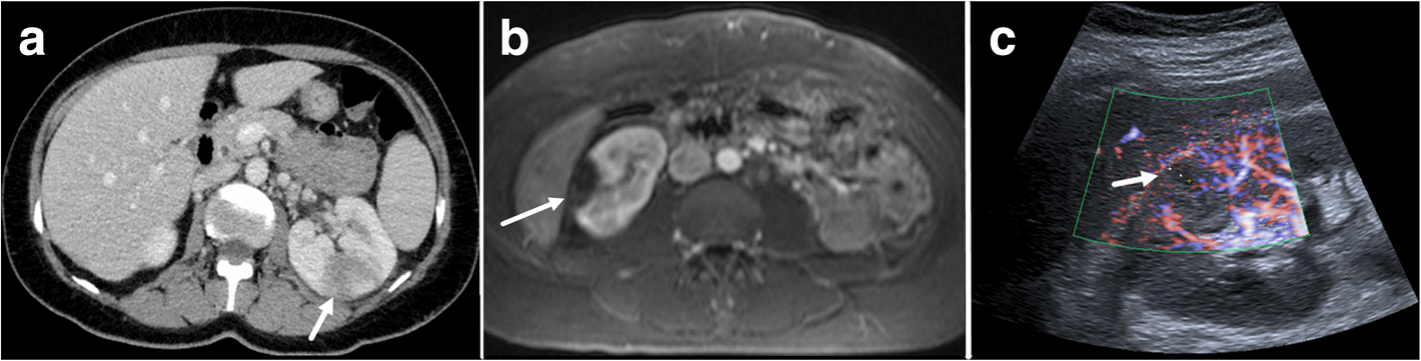
Imaging findings for AFBN. **a**-**c** Typical image findings for AFBN in CT (**a**), MRI (**b**) and Ultrasound (**c**). Post-contrast axial CT and MRI (T1w VIBE) showing areas of poor enhancement in the right kidney (arrows) in wedge (**a**) and round shape (**b**). Colour Doppler enhanced Ultrasound image (**c**) with arrow indicating focal hypoperfusion

Acute focal bacterial nephritis was first described in 1978 by Rosenfield et al. [1]. It was then named “acute lobar nephronia”, in analogy to acute lobar pneumonia, as the anatomic extent of the infection is sometimes determined by the renal lobes. AFBN is a rare focal bacterial interstitial infection of the kidney presenting with characteristic focal lesions in radiological imaging (Fig. 1). Focal lesions seen in AFBN are occasionally misdiagnosed and patients are at risk to receive unnecessary invasive procedures [1–4]. Given that AFBN is poorly described in the literature as available information predominantly consists of case reports and small case series, in this work, we conducted a systematic review of the literature in an attempt to summarize clinical presentation of the disease and make recommendations on its management.

## Methods

A systematic review of the literature on AFBN was conducted on PUBMED, Web of Science and The Cochrane Library online databases on July 2014. Search terms used were (focal[Title/Abstract]) AND *nephritis[Title/Abstract] in PUBMED and focal AND *nephritis (Title) in Web of Science and focal AND *nephritis (Title, Abstract, Keywords) in The Cochrane Library. We applied the Preferred Reporting Items for Systematic Reviews and Meta-analysis (PRISMA) flow diagram [5]. After eliminating duplicates, we screened publications by their title. Two independent authors (NS and IK) assessed abstracts and full texts of selected publications for the following eligibility criteria: Articles on AFBN published in english, spanish, italian, french and german; we excluded publications not written in Roman letters. Articles on AFBN in human adults; we excluded articles on pediatric cases apart from four case series reporting mixed children’s and adult’s cases. Articles providing at least information about clinical presentation, radiologic findings, therapeutic procedures and course of the disease; radiologic characteristics were to indicate AFBN. Disagreements were resolved by discussion and consensus.

A standardized form was used to extract the following data from articles: study design; patient age; gender; symptoms; presence of leukocytosis; urinalysis; pathogen identified by urine and/or blood culture and/or biopsy; previous illness; genitourinary tract abnormalities; type of diagnostic imaging and findings; therapy; choice of antibiotic; invasive procedures; course of disease; follow up.

An update of search was performed in November 2015 revealing no additional studies that fulfilled our inclusion criteria.

## Results

### Article selection

**Fig. 2:**
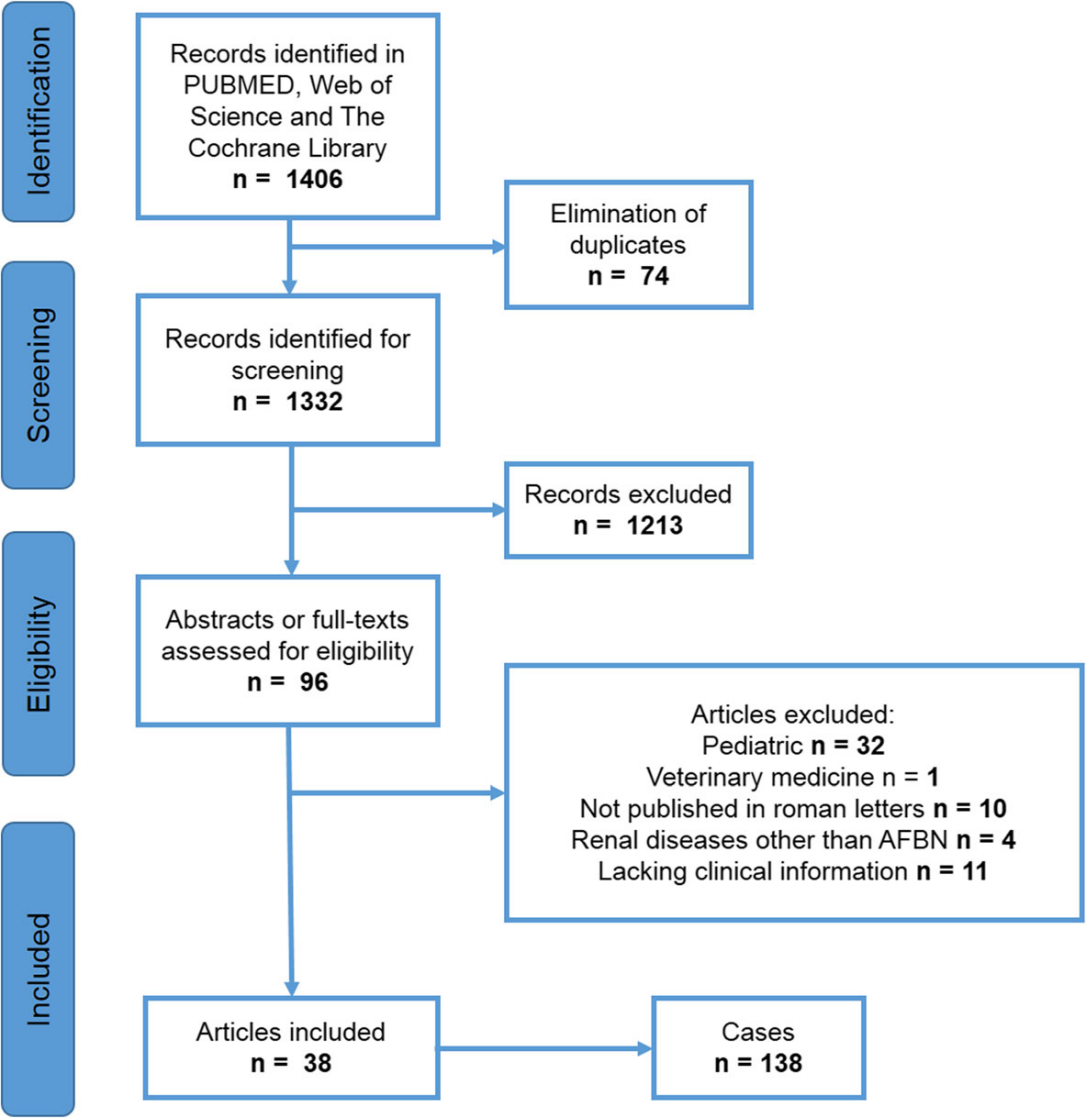
PRISMA Flow chart - Selection of publications

We included 38 publications according to our selection criteria. Out of these, we were able to extract clinical data from a total number of 138 cases (Table 1). The flow chart of our search strategy and article selection is presented in Fig. 2.

**Table 1:** Selected publications

Year	Author (et al.)	Number of cases (age, sex)
1979	Rosenfield	13 (1–66, f = 6, m = 6, not specified =1)
1980	Lee	13 (24–82, f = 8, m = 5)
1981	McDonough	4 (26–49, f)
1982	Funston	3 (44–52, f)
1983	Angulo	1 (31, m)
1984	Dochy	1 (35, f)
1985	Mc Coy	1 (43, f)
1985	Zaontz	9 (20–86, f = 8, m = 1)
1986	Rigsby	5 (17–40, f = 4, m = 1)
1986	Schmidt	5 (16–35, f = 3, m = 2)
1988	Cox	1 (19, f)
1988	Derouet	1 (22, m)
1988	Harpole	1 (57, f)
1988	Nosher	12 (5–62, f = 9, m = 3)
1990	Cuenca	1 (67, f)
1991	Dourthe	1 (26, m)
1992	Harris	1 (34, f)
1992	Levy	1 (33, f)
1992	Sawamura	2 (35–40, m)
1993	Thomalla	1 (22, m)
1994	Cho	24 (7–78, f = 20, m = 4)
1994	Ruiz Dominguez	1 (40, f)
1994	Yang	1 (42, m)
1995	Boam	1 (75, m)
1995	Wood	1 (73, m)
1996	Li	15 (16–56, f = 5, m = 10)
1996	Pelage	2 (24–31, f)
1996	Rosi	3 (11–43, f)
2000	Kumar	1 (44, m)
2001	Esteban	1 (43, f)
2001	Falcon	1 (37, m)
2002	Ameur	1 (24, m)
2002	Montejo	4 (20–65, f)
2004	Joss	1 (28, f)
2011	Čustović	1 (52, f)
2013	Adams	1 (33, f)
2014	Iga	1 (50, m)
2014	Maeshiro	1 (23, f)

### Clinical findings in AFBN

#### Fever and flank pain are leading symptoms

**Fig. 3:**
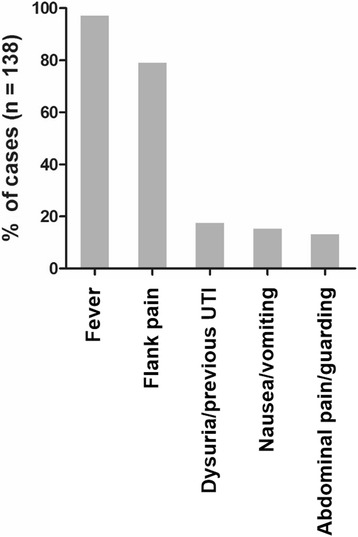
Symptoms in AFBN

AFBN is commonly presented with fever (reported in 98% of the cases) and leukocytosis, indicating severe illness and sepsis. Similar to acute pyelonephritis, ipsilateral flank pain is commonly encountered (80%), while dysuria and/or symptoms of lower urinary tract infection (UTI) were reported only in 18% of the cases in our cohort. Non-specific symptoms such as nausea, vomiting and abdominal pain or guarding may be present complicating differential diagnosis (Fig. 3). Palpation of an abdominal mass was reported in 7% of cases (*n* = 10) [3, 6, 7].

#### *E. coli* is the predominant pathogen

**Fig. 4:**
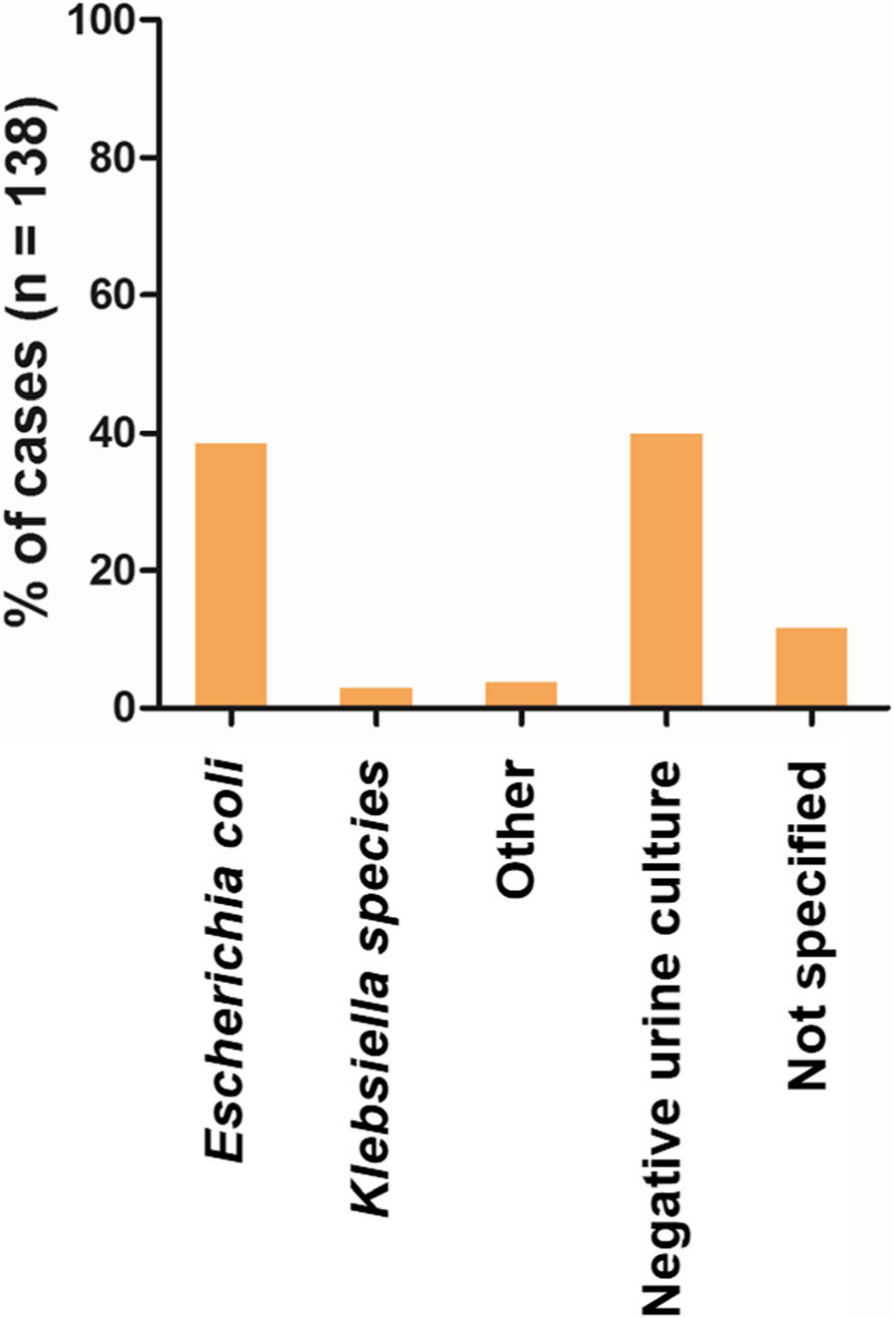
Urine microbiology in AFBN

**Fig. 5:**
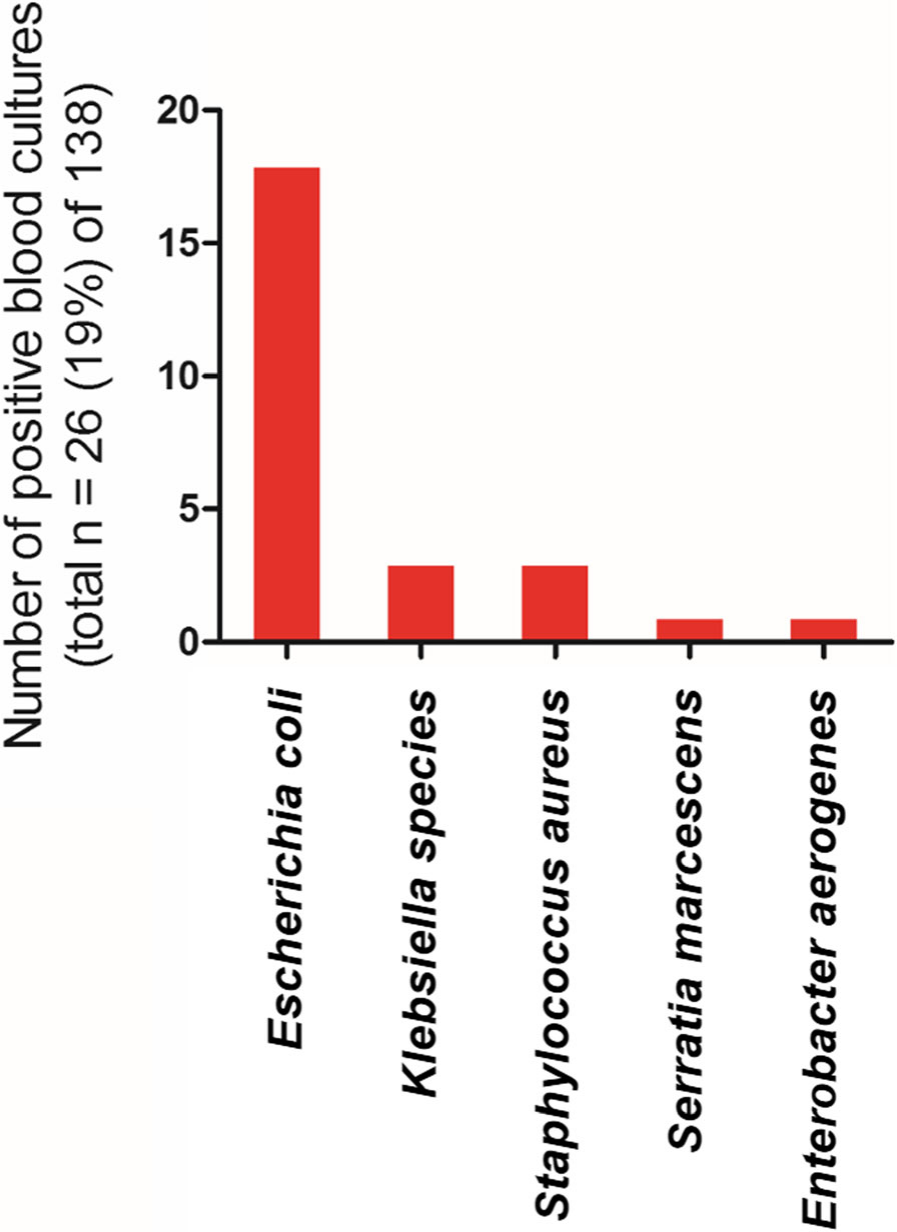
Positive blood cultures

The most frequent pathogen isolated in urinary cultures of patients with AFBN is *Escherichia coli*. In our cohort it was detected in 83% of all positive urine cultures. Sporadically, other gram negative bacteria such as *Klebsiella species* [1, 8, 9], *Proteus mirabilis* [10] and *Serratia marcescens* [11] have been isolated. Urine cultures were negative in 41% of cases. A summary of urine microbiologic findings is depicted in Fig. 4. Blood cultures were positive in 19% of cases and were negative or not specified for the rest. *E. coli* was also most commonly isolated in blood cultures representing 69% of positive blood cultures (Fig. 5). It is of worth mentioning that in 2.2% of the cases (*n* = 3) there was a strong indication of hematogenous spread. *Staphylococcus aureus* bacteremia was present in all these cases with concurrent AFBN and skin abscess [12], osteomyelitis [8] or glenohumeral septic arthritis [13]. One of the latter cases developed a renal abscess [12].

#### No underlying illness in the majority of cases

AFBN occurs more often in women than in men (ratio 2:1 in our cohort) and affects all ages from childhood to the elderly patient. Immune compromising diseases as well as other predisposing factors are associated with the formation of AFBN according to our cohort, such as Diabetes mellitus [1, 8, 9, 13–18], pregnancy [4, 14, 19], urinary tract abnormalities [1, 3, 4, 8–10, 14, 20, 21], prior or concurrent respiratory tract infection [3, 4, 16], former kidney transplantation [7, 22–24], alcohol and drug abuse [2, 25, 26], autoimmune diseases [1, 6, 10] and AIDS [25] (Table 2). Yet it has to be emphasized that AFBN also affects previously healthy immunocompetent patients.

**Table 2:** Predisposing illness and factors in patients with AFBN

	% (n)
Diabetes mellitus	12.3 (17)
Pregnancy	5.8 (8)
Renal stones without obstructive uropathy	5.1 (7)
Vesicoureteral reflux	3.6 (5)
Prior respiratory tract infection	4.3 (6)
Former kidney transplantation	2.9 (4)
Obstructive (nephro-)ureterolithiasis	1.4 (2)
Alcohol abuse	1.4 (2)
Neurogenic bladder dysfunction	1.4 (2)
Crohn’s disease	1.4 (2)
Prostatic hyperplasia	(1)
Lupus erythematodes	(1)
AIDS	(1)

### Diagnostic imaging

#### CT and sonography are preferred for diagnosis of AFBN

The diagnosis of AFBN is dependent on radiologic imaging. AFBN is characterized by hypoperfused wedged-shaped or round and space-occupying lesions in the kidney, exhibiting no capsule. Lesions can be uni- or multifocal. In our cohort 52% of patients received computed tomography (CT) and/or magnetic resonance imaging (MRI) during acute phase of AFBN all of them showing characteristic focal lesions. MRI was applied only in 2.9% of cases. Sonography in addition to CT and/or MRI was performed in 41% and had 91% sensitivity in this subgroup. In total, sonography was applied in 83% of cases (solely or combined with intravenous urography (IVU) and/or CT and/or MRI). Relating to the total amount of sonographies this diagnostic modality had 96% sensitivity in our cohort. 20% of the diagnoses were solely set by sonography. Radiologic features are summarized in Table 3.

**Table 3:** Radiological characteristics for AFBN

General characteristics	Specific features		
	CT	MRI	Sonography
Wedged-shaped or round	Hypodense in native scan	Hypointense in T2w	Hypo-, hyper- or isoechogenic
		Isointense in T1w	
Uni- or multifocal		Decreased contrast enhancement in T1w	Hypoperfused in Doppler
Non-liquefactive	Decreased contrast enhancement	Diffusion restriction	
Poor enhancing			
No capsule			

In native **CT** lesions are hypodense, showing decreased enhancement after intravenous application of contrast agent in comparison to normal parenchyma [3, 4, 8, 9, 27, 28]. They do not show cortical rim sign and thus wedged-shaped lesions can be differentiated from renal infarction [29]. Associated perinephric fat stranding might be present [12, 30]. The absence of contrast enhancement at lesion margins and the non-liquefactive density may enable the differentiation to renal abscess formation.

**MRI** was not often employed for AFBN according to the literature. In MRI focal lesions are hypointense in T2w, showing decreased enhancement in post-contrast T1w images [19, 31–33]. Relying data on diffusion weighted imaging is lacking, but lesions commonly show a certain degree of diffusion restriction in correlation to histopathological findings with high levels of cell-density in affected parenchyma.

In **sonography** focal lesions can be either hypo- or hyperechoic [10, 34, 35]. Doppler ultrasound with or without contrast enhancement confirms decreased focal blood flow [36]. In several cases there were no sonographic abnormalities while AFBN lesions were evident in CT [6, 18, 30, 34, 37, 38].

**Arteriographic** data on AFBN are available from publications from 1979 to 1992 confirming focal hypoperfusion. Application of this invasive technique was not mentioned beyond 1992. Similarly, employment of **IVU** for AFBN was last reported in 2002 [39]. IVU might suggest a renal mass with no or delayed contrast excretion or rather lack abnormalities [27, 33, 40].

### Histopathology

Histopathological data on AFBN were obtained by renal biopsies and (partial) nephrectomies. Focal lesions correspond to a zone of disturbed blood flow due to interstitial edema and perivascular inflammatory cells as well as mononuclear cells obstructing the veins. The formation of micro-abscesses might be present but there is no drainable pus [1, 2, 7, 17, 23].

### Treatment and interventions

#### Antibiotic therapy is the treatment of choice

All but one patient in our cohort received antibiotic treatment. The latter case was suspected for having a renal tumor and was directly treated with nephrectomy [3]. There is no standard regimen for choice of antibiotic agent and duration of antimicrobial therapy. The choice of antimicrobial agents was not specified in 82% of the cases. If specified, cephalosporins were most commonly used followed by broad-spectrum penicillins. Duration of treatment and hospitalization was very variable and not precised in most of the case reports. Duration of antibiotic treatment ranged up to 6 weeks. An uneventful course was reported after conservative management with antibiotics in the vast majority of cases. Still, treatment failure requiring additional measures was also reported. Four patients (2.9%) developed a renal abscess, three of which required drainage [11, 12, 26, 28]. One of the latter exhibited methicillin-resistant *Staphylococcus aureus* (MRSA) bacteremia. Two patients (1.5%) underwent percutaneous puncture due to persisting fever. Aspirates showed no drainable pus but grew *Pseudomonas aeruginosa* [23] and MRSA [30] respectively and adaptation of antibiotic therapy led to a favorable disease course for both patients. Two patients (1.5%) required ureteral stenting or stone extraction due to underlying obstructive lithiasis [4, 16] and three patients (2.2%) underwent nephrectomy because of reported non-response to antibiotic therapy or rather therapeutic mismanagement [1]. In addition to the above-named interventions further invasive procedures were applied in a significant number of cases due to uncertainty in diagnosis of AFBN, most commonly suspicion for a renal malignancy or abscess. A summary of all invasive procedures is shown in Table 4.

**Table 4:** Treatment and interventions

Treatment/Invasive procedures	% (n)	References
(Intravenous) antibiotic therapy	99.3 (137)	Table 1 (all)
Arteriography	13.8 (19)	[1, 4, 8–10, 16, 17, 27, 43, 44]
Percutaneous puncture	12.3 (17)	[2, 7–9, 12, 22, 23, 26–28, 30, 44]
Surgical exploration	5.1 (7)	[4, 8, 17, 28]
Partial or radical nephrectomy	4.4 (6)	[1–4]
Ureteral stenting/stone extraction following ureterolithiasis	1.5 (2)	[4, 16]

#### Relapses were rarely reported

Clinical relapse after initial treatment was reported in 2.9% of cases (*n* = 4). Three out of the four patients recovered upon conservative re-treatment [1, 8]. One patient exhibiting poor general state of health and noncompliance with the ongoing oral medication after discharge returned septic and died [28].

### Follow-up

Follow-up was not systematically performed according to the literature. Range of follow-up varied greatly from a short period to up to 4 years after disease onset and was performed by sonography, IVU, CT or MRI. There is evidence that radiologic residues of AFBN are frequently detected between ten days to 4 weeks after disease onset despite of reduction of symptoms. Four to eight weeks after disease onset the majority of reported radiologic investigations revealed no residues. Still, late renal scaring has been documented in 4.4% of cases (*n* = 6). CT [38], sonography [1, 10, 23] and IVU [28] in those cases showed focal parenchymal wasting or increased echo but patients were asymptomatic and no clinical relevance was reported to be related to these findings.

## Discussion

While AFBN was first described in 1978, it wasn’t until the establishment of CT and MRI as standard radiologic investigations in the following years, when a more detailed imaging of the kidney made AFBN a distinct clinical entity. Nevertheless, its diagnosis and management remain a challenge for clinicians and radiologists, as evidenced by the frequent application of redundant invasive interventions, due to its infrequent incidence. The aim of our study is to summarize accumulated knowledge by grouping the numerous case reports into a single large cohort of patients. Obviously, such analysis entails limitations that should be taken into consideration during interpretation of our results. As our dataset is dependent on individual authors´ reports, a selection and reporting bias is present; AFBN cases undergoing an uneventful course are less likely to be reported than cases with adverse outcomes; overtherapy (e. g. nephrectomy) in a case of AFBN is not commendable and therefore less likely to be reported.

AFBN may be underdiagnosed. Based on the presence of clinical symptoms of upper UTI only (fever and flank pain with or without dysuria) AFBN cannot be distinguished from acute pyelonephritis (AP). Sonography has shown high sensitivity (91–96%) in our cohort but there is evidence that CT and MRI are superior in diagnosing AFBN [18, 34, 37]. High sensitivity of ultrasound might be due to a selection bias (AFBN cases showing evident characteristic pathologies in ultrasound are more likely to be reported). As a result, if CT scan was performed in every patient exhibiting signs for upper UTI, AFBN would probably be diagnosed more frequently. AP can also present with radiological signs (renal enlargement, (diffuse) disturbance of perfusion in renal parenchyma) but in contrast to AFBN radiologic evidence is not mandatory for diagnosis of AP. As soon as localized uni- or multifocal lesions are detected the term acute focal (or multifocal) bacterial nephritis should be used implicating a higher risk for complicated disease course and redundant interventions. It is not relevant to distinguish all cases of AFBN from AP but to identify serious and atypical courses of AFBN requiring special attention and prolonged treatment.

The pathophysiological mechanism of AFBN remains unclear. Hematogenic spread might play a particular role in this disease as the localization of focal lesions seem to correspond to the blood supply pattern of renal segmental arteries, suggesting that focal renal infections are caused by dissemination of bacterial emboli [3]. Presence of a known source of distant infection (skin abscess, osteomyelitis, glenohumeral septic arthritis) was associated with *Staphylococcus aureus* bacteremia. Some authors suggest that AFBN is a transitional stage between diffuse pyelonephritis and abscess. Indeed, a shift of AFBN to renal abscess has been described in 2.9% of our cases [11, 12, 26, 28], indicating that an abscess was the result of an insufficiently treated AFBN. Presence of microabscesses has been histologically confirmed in several AFBN specimens. In borderline stages it might be difficult to distinguish AFBN from an abscess by radiologic imaging. Yet, this discrimination is very important as AFBN does not require drainage (pus evacuation has never been reported) in contrast to an abscess. Normally AFBN exhibits diminished uptake of contrast agent, while an abscess is not perfused.

### Recommendation on diagnostic, therapy and follow-up

Sonography should be applied as initial screening method in all patients with upper urinary tract infection [41]. It is not sensitive enough to clarify AFBN in all cases but is helpful in detecting mass-like lesions in severe cases of AFBN, renal abscess and other renal pathologies. Doppler ultrasonography in AFBN reveals focal hypoperfusion and allows distinction from an abscess. If there is remaining dubiety and severe clinical illness, contrast enhanced CT scan can elucidate the focal pattern of kidney lesion and exclude other causes of acute abdomen. Despite the underutilization of MRI in our current cohort, the technique is an alternative to CT with similar sensitivity.

Several authors reported non-response of AFBN to oral treatment in the pre-hospital setting and necessity for an escalation to intravenous antibiotics [13, 15, 21, 22] and hospitalization. As a result, intravenous administration of antibiotics should be recommended. For empiric therapy it should be taken into account that *E. coli* and other gram-negativ bacteria are the most frequent pathogens causing AFBN. The local infective guidelines for urinary tract infections should be considered. There is evidence that fever and disease course last longer in AFBN than in AP [37]. As a result, intravenous therapy should be continued for at least two days after defervescence and changed to oral antibiotics not before reduction of symptoms. We recommend to continue oral antibiotic intake in the post-hospital setting for at least 2 weeks and not to stop before resolution of pain. Exact time span for oral medication should be chosen depending on the course of the disease and taking into consideration that follow up examinations have shown delay of complete resolution of focal lesions for up to several weeks. Especially multifocal mass-like lesions in radiologic imaging have shown to correlate with a prolonged and complicated clinical course compared to single wedge-shaped lesions [42].

In contrast to the management of renal abscess, no drainage is primarily indicated since there is no drainable pus in AFBN. Yet, percutaneous puncture might be helpful in patients whose condition is worsening despite antibiotic therapy. In these cases cultivation of the aspirate can help to directly identify pathogen and adapt antibiotic therapy. Also, it should be taken into account that AFBN can very rarely turn into an abscess despite antibiotic therapy, especially when the pathogen is *Staphylococcus aureus*.

**Fig. 6:**
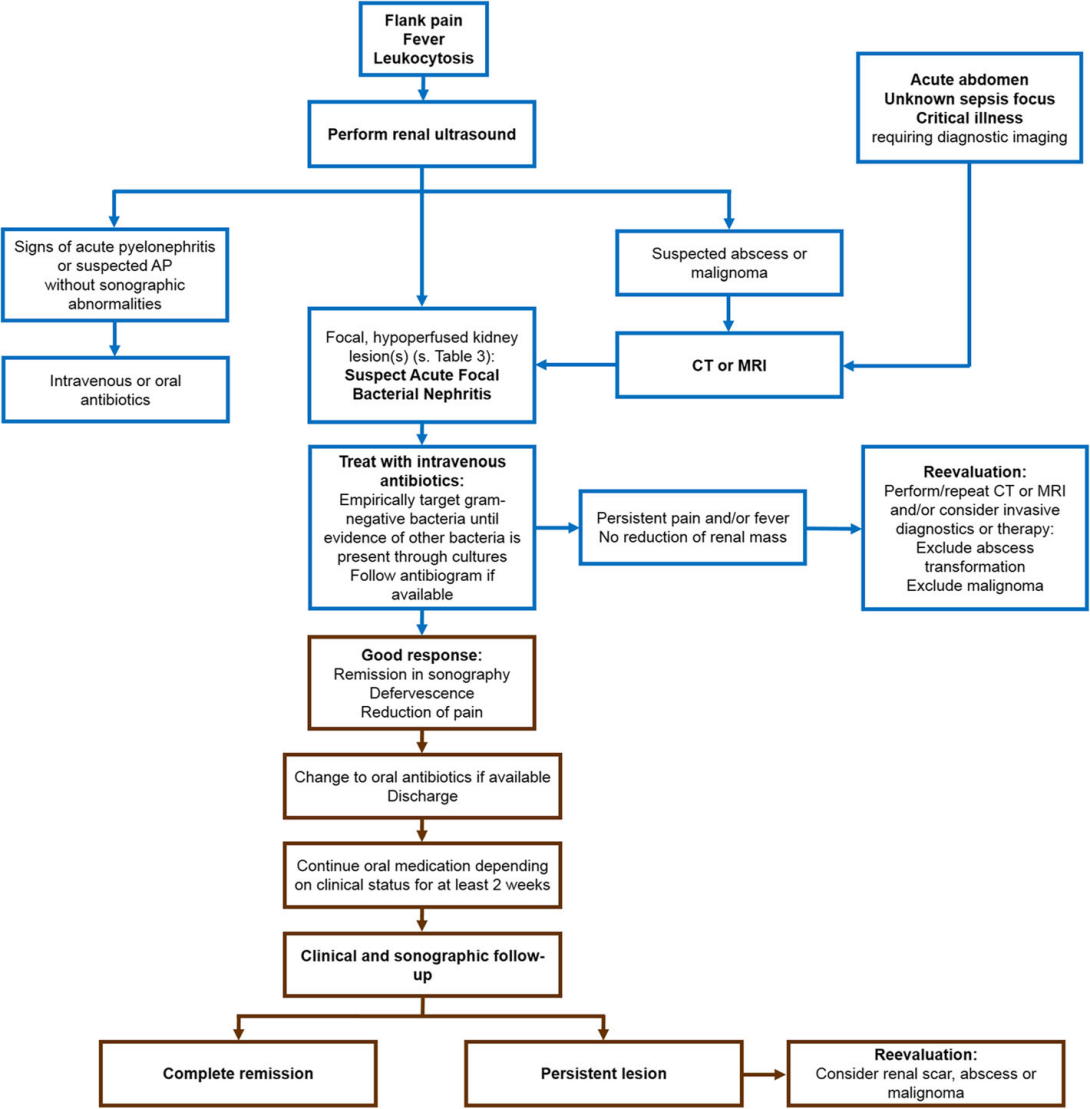
Diagnostic and treatment algorithm for AFBN

Since patients mostly respond well to conservative treatment we do not recommend a general follow-up regime that involves radiation exposure. We suggest sonography for follow up in case of proper resolution of focal lesions and uncomplicated disease course without clinical evidence for persistent renal infection. An overview of our recommendations is shown in Fig. 6.

## Conclusions

Correct interpretation of both clinical and radiological findings is crucial for diagnosing AFBN. Differential diagnoses include renal abscess and tumor as well as other infective diseases. A significant number of invasive procedures has been reported in the management of AFBN including percutaneous puncture, surgical exploration and even partial or radical nephrectomy. The majority of such interventions could be considered redundant, as AFBN is a kidney infection shown to be reversible upon antibiotic treatment.

## Abbreviations

AFBN: Acute focal bacterial nephritis
AIDS: Acquired immunodeficiency syndrome
AP: Acute pyelonephritis
CT: Computed tomography
*E. coli*: *Escherichia coli*
f: Female
IVU: Intravenous urography
m: Male
MRI: Magnetic resonance imaging
MRSA: Methicillin-resistant *Staphylococcus aureus*
PRISMA: Preferred Reporting Items for Systematic Reviews and Meta-analysis
UTI: Urinary tract infection
